# Hip arthroscopy in obese, a successful combination?

**DOI:** 10.1093/jhps/hnv076

**Published:** 2015-11-27

**Authors:** N. H. Bech, I. F. Kodde, F. Dusseldorp, P. A. M. C. Druyts, S. P. L. Jansen, D. Haverkamp

**Affiliations:** 1. Department of Orthopedic Surgery, Slotervaart Medical Center, Louwesweg 6, Amsterdam 1066 EC, the Netherlands; 2. Department of Orthopedic Surgery, TweeSteden Hospital, Dr Deelenlaan 5, Tilburg 5042 AD, the Netherlands; 3. Department of Orthopedic Surgery, Rijnland Hospital, Simon Smitweg 1, Leiderdorp 2353 GA, the Netherland

## Abstract

Discussion persists about the outcome and results of hip arthroscopy in obese patients. Hip arthroscopy gained popularity over time. A current discussion is if obese patients can reach similar results after surgery compared with non-obese. To our knowledge, this is the first systematic review of literature about hip arthroscopy and obesity. We searched the Pubmed/Medline databases for literature and included three studies that compared the outcome of hip arthroscopy between different BMI groups. We extracted and pooled the data. For continues data a weighted mean difference was calculated, for dichotomous variables a weighted odds ratio (OR) was calculated using Review Software Manager. Heterogeneity of the included studies was calculated using I^2^ statistics. Data were extracted from two studies. In the Obese group, there was significant more conversion to total hip replacement or resurfacing hip replacement (OR = 2.21, 95% CI 1.07–4.56) and more re-arthroscopy (OR = 4.68, 95% CI 1.41–15.45). Any reoperation occurred more often in the obese group (OR = 2.87, 95% CI 1.53–5.38). In the Non Arthritic Hip Score obese scored lower than the non-Obese group [10.9 (−14,6 to 7.1)]. For the modified Harris Hip Score the score is − 6,6, according to the MCID this difference is clinically relevant. For both scores obese show lower outcomes but similar improvement after hip arthroscopy. Regarding a higher chance of needing a re-operation and lower subjective outcome scores obesity appears to have a negative influence on the outcome of hip arthroscopy.

## INTRODUCTION

The clinical applications of hip arthroscopy evolved mainly last decade. An important indication for hip arthroscopy nowadays is the management of symptomatic FAI and labral tears. Surgical treatment of FAI aims to improve symptoms, increase function and prevent the possible progression to end stage hip osteoarthritis and total hip arthroplasty.

A possible risk factor to develop osteoarthritis of the hip is obesity. A review performed by Jiang *et al.* [1] shows that an increased body mass index (BMI) contributes to a positive effect on susceptibility of hip osteoarthritis. Obesity is a worldwide health problem with more than 1.9 billion adults (18 years and older) being overweight and 600 million of these people being obese [2]. Overweight is classified as a BMI >25 kg/m^2^ and obesity as a BMI >30 kg/m^2^.

An MRI study performed by Teichtahl *et al.* [3] showed that obesity is associated with deformities of the acetabulum, especially increasing acetabular depth. This is associated with reduced femoral head cartilage and might be an explanation of the increased risk of hip osteoarthritis in obese, or predisposing impingement. The development of osteoarthritis in obese patients may depend more on their weight, rather than an FAI problem.

Clohisy *et al.* [4] recently studied the epidemiology of surgical interventions for symptomatic FAI, and showed that almost 42% of patients operated for FAI are overweight or obese. A program existing of exercises and weight loss has been shown to be a successful treatment for patients with hip osteoarthritis and overweight or obesity [5]. It is also debatable whether obese patients can really develop FAI as the range of motion of obese patients for flexion and internal rotation is limited [6].

It has been recognized that there is a correlation between obesity and various joint complaints [7]. A study performed by Rajämaki *et al.* [8] showed that patients with diabetes have more persistent joint pain after knee or hip surgery. It is known that diabetes is more common in obese than non-obese patients therefore it is understandable that obese patients might suffer persistent joint pain after hip arthroscopy.

The purpose of this study was to systematically review the literature on the outcomes of hip arthroscopy in obese patients compared with non-obese. Our hypothesis was that obese patients profit less of hip arthroscopy, have more complications and have worse subjective patient reported outcome measures.

## METHODS

A research protocol was developed as described by Wright *et al.* [9] and used throughout the study process. This protocol was not registered. A literature search was performed through the Pubmed/Medline databases on the 26 March 2015. The following Mesh terms were used: (obesity OR body weight OR Body Mass Index) AND (hip AND arthroscopy). Furthermore, the lists of references of retrieved publications were manually checked for additional studies potentially meeting the inclusion criteria but not found by the electronic search. Two investigators independently reviewed the literature to identify relevant articles for full review. From the full text, using the above-mentioned criteria, the reviewers independently selected articles for inclusion in this review. Studies were included if they were comparative trials comparing the outcome of hip arthroscopy between different BMI groups. Review articles, expert opinions, surgical techniques and abstracts from scientific meetings were excluded. Only articles written in English were included. Studies were not blinded regarding author, affiliation or source. This systematic review and meta-analysis were done according to the PRISMA guidelines.

Our primary research question was to determine whether the outcome of hip arthroscopy is influenced by BMI. Our outcomes were complications; patient-reported outcome measures, reoperation rates and conversion rates into arthroplasty.

### Statistics

One reviewer using a pre-piloted data extraction tool extracted the data from the studies included, and the second reviewer verified them. Then the available data from the selected studies were pooled using the Review Manager software from the Cochrane Collaboration. For outcome variables with a continuous nature, a weighted mean difference was calculated with 95% confidence interval (CI). For the dichotomous variables, a weighted odds ratio (OR) with 95% CI was calculated using Review Manager software.

For the studies where continuous variables were reported with a range, the SD was calculated. The heterogeneity of the studies included was calculated using I^2^ statistics. This measurement describes the percentage of variation across studies, which is due to heterogeneity rather than chance [10]. We also assessed heterogeneity by means of a Chi-square analysis, whereby a *P* value of <0.1 was considered to be suggestive of statistical heterogeneity.

## RESULTS

Three studies were identified, of which two are from the same author. Gupta *et al.* [6, 11] reported in 2015 in two articles on a patient population that is operated on by the same senior author in the same time period, we therefore suspect that both are the same population and included only the larger of the two series. We included the larger cohort analysis with 562 patients in the non-obese group, 94 patients in the class 1 obese group and 24 in the class 2 obese group. In Collins *et al.*’s [12] study, 39 patients were enrolled, 18 non-obese and 21 in the obese group.

In Collins study, there was no significant difference in demographics, in Gupta’s study the class 1 obese group was significantly older than the non-obese group.

**Fig. 1:**
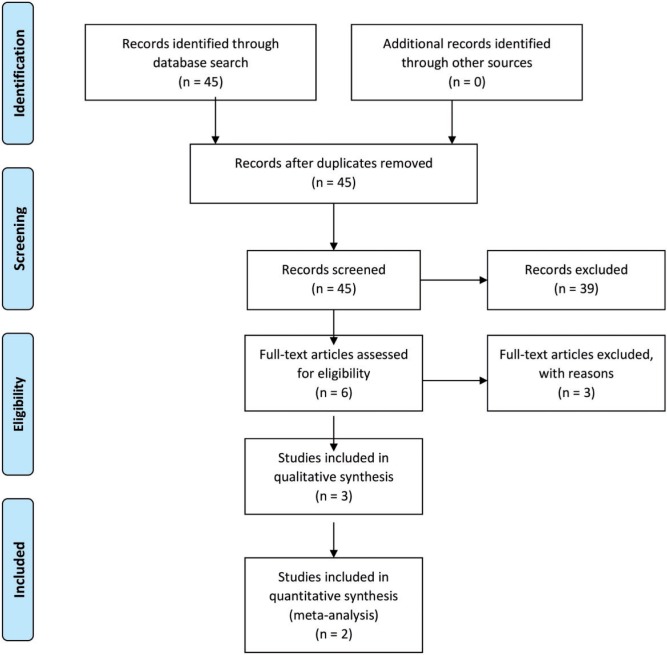
Prisma flow chart.

Both included studies report of an average of 2.5 years follow up (Fig. 1). For analysis purposes we combined the Class 1 and Class 2 obesity groups from the Gupta study into one group defined as obese to allow pooling with the second study of Collins *et al.* [6, 12].

**Fig. 2:**
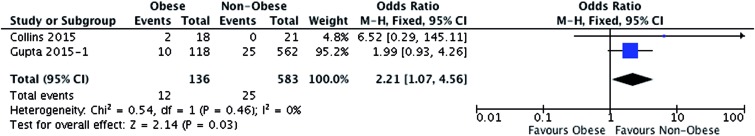
Conversion to THR or resurfacing hip prosthesis.

**Fig. 3:**
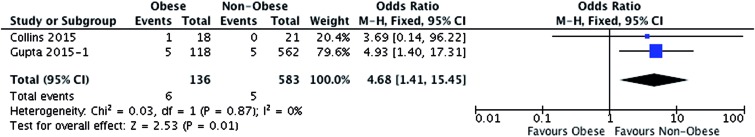
Re-arthroscopy rate.

**Fig. 4:**
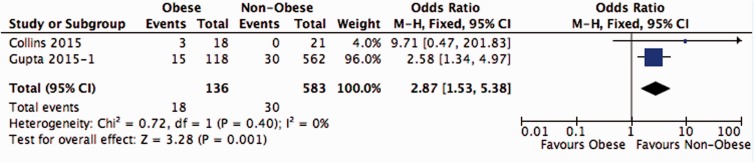
Conversion to THR or resurfacing hip prosthesis and re-arthroscopy rate combined.

Conversion to THR (or resurfacing) shows an OR of 2.2 (1.1–4.6) in favour of the Non-Obese (Fig. 2). Re-arthroscopy can be defined as failure of previous arthroscopic surgery, showing a pooled OR of 4.7 (1.4–15.5) in favour of the Non-Obese group (Fig. 3). Any reoperation on the same hip shows an OR of 2.9 (1.5–5.4) (Fig. 4).

**Fig. 5:**
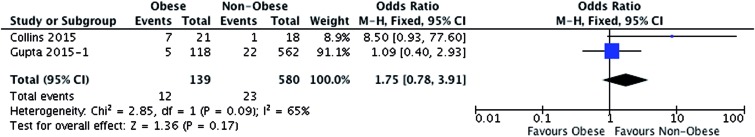
Complications.

When comparing the complications between the groups, no significant difference was found [OR 1.8 (0.8–3.9)], pooled complications rate are 4% in the Non-Obese group and 9% in the obese group (Fig. 5).

Subjective outcomes in obese are lower than in the non-obese population. For the modified Harris Hip Score (mHHS) this score is below 80 in the obese population, which is classified in the original HHS publication as a fair outcome [13]. The difference after pooling [−6.6 (−10.2 to −2.9)] is more than the MCID for the HHS, being 4, therefore it can be said that the outcome difference is clinically relevant.

**Fig. 6:**
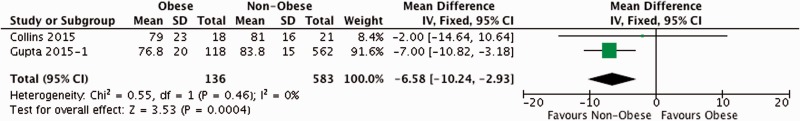
Harris Hip Score.

The Non Arthritic Hip Score (NAHS) in which the obese score 10.9 lower than the Non-Obese (−14.6 to −7.2) (Figs. 6 and 7).

In Gupta’s series, the non-obese start at significantly higher patient reported outcomes at baseline. All three groups show similar significant improvement postoperatively. There was no significant difference in change of patient reported outcomes between the non-obese group and either one of the obese groups.

In Collins series, both groups showed a statistical significant improvement from baseline for as well the NAHS as the mHHS. There was no significant difference between baseline and change in the NAHS and the mHHS between the obese and non-obese.

## DISCUSSION

Our systematic review shows that the results of hip arthroscopy in obese might have a poorer outcome. Although obese patients show similar improvement after surgery, subjective outcome scores are lower at follow up and re-arthroscopy rates are 4.7 times higher and conversion to hip replacement 2.2 times higher for obese.

The main concern is that two recent publications by the same group conclude that the outcome of hip arthroscopy in obese is comparable to that in non-obese. However, the included study of Gupta has some flaws. First, reoperation rate is divided in re-arthroscopy, total hip replacement (THR) and resurfacing and all these comparisons do not reach significance. Dividing resurfacing and THR in different groups is in our opinion not correct since both are hip replacement surgery.

The main shortcoming of our review is that only two studies were suitable for inclusion, though both were comparative studies of prospectively followed patients. However, we feel that our review is an important addition to the current knowledge. A quick reader may conclude that there are three studies showing non-inferior results in obese, while the result in obese may indeed be inferior compared to non-obese.

In Gupta’s series, there was a higher percentage of patients in the obese Class 1 and Class 2 groups who went for revision arthroscopies. This could be due to the higher percentage of capsular release in these groups (70.2 and 62.5%).

Both included studies used for the meta-analysis are performed in the USA where 68% of the general adult population is obese [14]. There are several other confounding factors associated with different BMI in the USA: socioeconomic class, income or race for example.

Patients with a high BMI more often origin from a lower socioeconomic class [14]. These patients have less financial means and if primary surgery fails or is inadequate, they might earlier opt for a definite solution; THR. Diabetes is more common in obese patients than non-obese. Rajämaki *et al.* [8] showed that diabetic patients suffer more postoperative pain after knee and hip surgery. This can be another reason that obese patients might earlier opt for hip replacement surgery. In retrospective studies comparing obese and non-obese, the presence of selection bias is indeed not unlikely. Besides that, the studies do not mention whether the patients were obese their whole life, or even if they were already obese when the complaints started, just the BMI at time of surgery is stated. By not knowing BMI change over time it is more difficult to judge several details of this group.

There is paucity in literature regarding obesity and arthroscopy of the hip but there is comparative literature from arthroscopic knee surgery. Erdil *et al.* [15] evaluated the results of more than 1000 patients who underwent knee arthroscopy for partial meniscectomy. They compared the effect of BMI on functional outcome and divided all patients in one of three groups; (1) normal weight (BMI <26 kg/m^2^), (2) overweight (BMI of >26–29.9 kg/m^2^) and (3) obese (BMI >30 kg/m^2^). Compared with the normal weight group, both the overweight as the obese group showed significant worse short-term outcomes using the International Knee Documentation Committee, the Lysholm Knee Scale and Oxford Scoring System scores.

Harrison *et al.* [16] compared the results of knee arthroscopy in overweight women versus normal weight women 4–11 years after surgery. In all domains of the SF-36 questionnaire, obese women showed significant lower outcome scores and were less satisfied.

It is quite understandable that performing hip arthroscopy on obese patients can be more challenging regarding patient positioning, portal placement and traction times. All these factors result in longer operative times and therefore can lead to more complications. In this study, there were not significant more complications in the obese population, however with an OR of 1.75 the non-significance may be caused by lack of power.

A study of Paans *et* *al.* [5] showed that with an 8-month program of physical therapy and weight loss, patients with degenerative hip complaints had an improvement of 33% on the WOMAC scale. In the included studies an improvement was reached in 2.5 years of 28% in the study of Gupta *et* *al.* and 43% in the study of Collins. In both studies, the duration of physical therapy and the weight change over time is not included in the analysis. It is not uncommon for hip arthroscopy patients to start vigorous rehabilitation programs after surgery in which weight reduction could be part of the goal. This might create a bias if not included in the final analysis.

A lot can be said over the cause of hip pain and the problems that can be solved with hip arthroscopy, and that clear indications might give good results even in the obese. The problem in analysing the data is that in both studies every hip arthroscopy, for every indication, is combined. In the study of Collins *et al.*, the pathology remains intra-articular, whereas in the study of Gupta *et al.*’s even extra-articular procedures like IT band release, sciatic nerve decompression and piriformis release are included. This heterogeneous group makes it impossible to state something about specific indications in combination with obesity and the possible outcome of hip arthroscopy.

## CONCLUSION

Hip arthroscopy in obese show similar improved results after surgery, but with lower overall outcome scores and more re-operations one can question if hip arthroscopy is the right option in obese patients. Since obesity itself can possibly be the causative factor, we advise caution with surgical interventions and focus more on weight loss programs with physical therapy prior to surgery.

## CONFLICT OF INTEREST STATEMENT

None declared.
